# Enhancing the use of research in health-promoting, anti-racism policy

**DOI:** 10.1186/s12961-017-0223-7

**Published:** 2017-07-11

**Authors:** Angeline S. Ferdinand, Yin Paradies, Margaret Kelaher

**Affiliations:** 1 Centre for Health Policy, Melbourne School of Population and Global Health, University of Melbourne, Melbourne, Australia; 2 Alfred Deakin Institute for Citizenship and Globalisation, Faculty of Arts and Education, Deakin University, Burwood, Australia

**Keywords:** Anti-racism, Health, Aboriginal, Cultural diversity, Knowledge translation, Partnerships, Interventions, Double-loop learning, Organisations, Implementation

## Abstract

**Background:**

The Localities Embracing and Accepting Diversity (LEAD) programme was established to improve the health of ethnic minority communities through the reduction of racial discrimination. Local governments in the state of Victoria, Australia, were at the forefront of LEAD implementation in collaboration with leading state and national organisations. Key aims included expanding the available evidence regarding effective anti-racism interventions and facilitating the uptake of this evidence in organisational policies and practices.

**Methods:**

One rural and one metropolitan local government areas were selected to participate in LEAD. Key informant interviews and discussions were conducted with individuals who had participated in LEAD implementation and members of LEAD governance structures. Data were also collected on programme processes and implementation, partnership formation and organisational assessments.

**Results:**

The LEAD model demonstrated both strengths and weaknesses in terms of facilitating the use of evidence in a complex, community-based health promotion initiative. Representation of implementing, funding and advisory bodies at different levels of governance enabled the input of technical advice and guidance alongside design and implementation. The representation structure assisted in ensuring the development of a programme that was acceptable to all partners and informed by the best available evidence. Simultaneous evaluation also enhanced perceived validity of the intervention, allowed for strategy correction when necessary and supported the process of double-loop organisational learning. However, due to the model’s demand for simultaneous and intensive effort by various organisations, when particular elements of the intervention were not functional, there was a considerable loss of time and resources across the partner organisations. The complexity of the model also presented a challenge in ensuring clarity regarding roles, functions and the direction of the programme.

**Conclusions:**

The example of LEAD provides guidance on mechanisms to strengthen the entry of evidence into complex community-based health promotion programmes. The paper highlights some of the strengths and weaknesses of the LEAD model and implications for practical collaboration between policymakers, implementers and researchers.

## Background

Gaps between the creation of knowledge and its incorporation into health programmes and policies stubbornly remain despite much consideration of the problem [1]. An approach that continues to hold promise is the creation of various structures that bring together researchers, funders, policymakers, implementers and other stakeholders in the hopes that the translation of evidence into policy and practice will be facilitated by these actors working closely and in parallel [1, 2]. That is, these partnerships aim to support the production of knowledge that aligns with the priorities and needs of decision-makers, while also increasing decision-makers’ access to available evidence. Partnerships and collaborations are a key theme in health promotion and there is a wide range of models in this field [3, 4]. One such model is Wenger’s Communities of Practice approach, which sees collective learning as a community of individuals that come together for a shared purpose and participate in improving practice through mutual engagement and sharing of resources [5]. When this community stretches across those who produce and those who utilise evidence, effective knowledge translation may result [6]. In the New Zealand context, partnerships as part of treaty-based health promotion practice has been identified as a way to promote research that is useable in policy and practice [7]. However, there remains a lack of systematic evidence regarding what types of partnership models are most effective in particular circumstances, the factors that support such effectiveness, and who should be involved in such partnerships and in what capacity [2, 8].

A disconnect between evidence and policy and practice can be seen in attempts to address racism and develop effective anti-racism interventions. While the factors contributing to racism are complex, there is increasing evidence that racism can be reduced, and that anti-racism interventions can contribute positively to health in minority populations [9, 10]. Williams et al. [9] argue that evidence points to promising and potential avenues for addressing disparities in health and improving health equity for people from minority backgrounds through the reduction of racism in a range of contexts, including healthcare, education and media. However, both the current body of evidence and appropriate use of existing evidence are insufficient to adequately inform the planning and implementation of anti-racism interventions [9].

Localities Embracing and Accepting Diversity (LEAD) was established by the Victorian Health Promotion Foundation (VicHealth) to improve health and reduce anxiety and depression among Aboriginal Australian and culturally and linguistically diverse (CALD) communities through the prevention of racism. The LEAD programme and evaluation was guided by the *Building on Our Strengths* framework [11]. The *Building on Our Strengths* framework incorporates an ‘evidence-informed’ approach in addressing both systemic and interpersonal racism, recognising that these are inter-related phenomena. The framework is primarily focused on preventing discrimination before it occurs. While it is designed to guide anti-discrimination initiatives affecting people from Indigenous backgrounds as well as those from migrant and refugee backgrounds, it also recognises the need to attend to the different factors influencing discrimination affecting these two sub-populations when specific interventions are being considered [11]. In line with *Building on Our Strengths*, the LEAD programme and evaluation took a strengths-based approach. As such, the experiences and knowledge of affected communities regarding racism were centred through representation on advisory committees, multiple avenues for community members to guide programme development and continuous feedback to communities throughout the programme and evaluation.

The structure of LEAD was underpinned by the need to address the lack of evidence regarding effective anti-racism interventions and the need to bring what evidence did exist to bear on the design and implementation of the programme. The key aims of LEAD therefore included generating high-quality evidence regarding anti-racism interventions through programme evaluation as well as facilitating the uptake of available evidence into the processes of policymaking and intervention development and implementation. As such, the LEAD evaluation utilised an action research approach. Trip [12] comments that the term has become used extremely diffusely, but characterises action research as “*employ*[ing] *recognised research techniques to inform the action taken to improve practice*”. LEAD evaluation was conducted alongside programme implementation, and evaluation findings and other available evidence were continuously fed back into developing future programme strategies.

This paper aims to explore the LEAD model in order to consider concrete mechanisms for structuring health programmes to enhance ongoing interactions between evidence and practice.

**Fig. 1:**
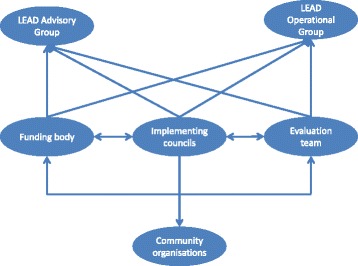
LEAD partnership model

LEAD was implemented as a place-based intervention between July 2009 and July 2013, with two councils being the primary implementing partners. ‘Councils’ refers to local governing bodies, with the areas governed by councils termed local government areas (LGAs). The governance structure of LEAD was designed to strengthen cohesion between implementation, process and outcome evaluation and policy impact. LEAD governance consisted of the LEAD Advisory Group and the LEAD Operational Group. Membership of the LEAD Advisory Group included senior representatives from VicHealth as the primary LEAD funding body, each of the implementing councils, the Victorian Equal Opportunity and Human Rights Commission, *beyondblue* (Australia’s peak body for mental health issues), the Municipal Association of Victoria (the peak body for councils in the state of Victoria), the Department of Immigration and Citizenship, and the University of Melbourne evaluation team. The LEAD Advisory Group met every 6 months and provided an opportunity for the partner and funding agencies and councils to guide the programme design. The Advisory Group was also designed to facilitate ongoing input from communities affected by racism, with representation from Aboriginal and CALD community groups. Local advisory committees at the council level also promoted close dialogues between Aboriginal and CALD community groups and the LEAD Project Officers. This enabled community members to stay up-to-date with project processes, have input into strategies and provide feedback on perceived outcomes, and allowed Project Officers to have more clarity around the perspectives of communities. Inclusion of members from both Aboriginal and CALD communities in advisory structures allowed a more nuanced understanding of the different ways these groups experience racism and their particular positions in Australian society. The LEAD Operational Group met approximately every 2 months and consisted of representatives from the councils, VicHealth, the Victorian Equal Opportunity and Human Rights Commission, Municipal Association of Victoria and the University of Melbourne evaluation team, and served to support networking and information exchange among organisations directly involved in LEAD implementation and evaluation. LEAD was also supported by the Lowitja Institute, Australia’s national institute for Aboriginal and Torres Strait Islander health research [13] (Fig. 1).

As the primary funding body, VicHealth managed the evaluation contract with Melbourne University and coordinated dissemination with other organisational partners. In addition, VicHealth worked with the councils – the main implementing partners – to develop strategies that were based on the best available evidence and relevant to their local contexts while maintaining some level of consistency across the two sites. These strategies were developed and conducted in partnership with local community organisations, including Aboriginal and CALD communities, schools, workplaces, retail settings and the councils themselves, with input from the evaluation team regarding available evidence. These partnerships included a number of facets. Wide-reaching consultation sessions were held with community organisations and groups to understand local contexts, factors and concerns. During these sessions, background on the aims and approach of LEAD was presented, while information was received regarding what had previously been trialled to address racism, the success of previous interventions, and ideas for future strategies. Ongoing CALD and Aboriginal community feedback and input into LEAD design and implementation was achieved through representation on advisory committees. Community evaluators were trained and employed to administer surveys regarding the experiences of racism within CALD and Aboriginal communities. Meetings, informal conversations and debriefing sessions with the community evaluators provided another avenue for input into programme design, while dissemination of survey findings through community events allowed for direct contact and information exchange between community members and the evaluation team. In addition to initial information and consultation sessions, regular meetings were held between participating organisations and the LEAD Project Officers, which allowed for ongoing feedback regarding the design, implementation and perceived success of the strategies undertaken in schools, workplaces, retail settings and the councils themselves.

A wide variety of approaches were trialled within these settings. Pro-diversity and cultural awareness training, human resources policy reform, organisational assessment of policies, practices and procedures, and internal communication and awareness-raising were most frequently employed [13].

While every organisation involved in LEAD espoused support for anti-racism ideals, baseline surveys demonstrated that employees experienced some level of discrimination [14]. Additionally, the practice of talking openly about race and racism or acknowledging the presence of racism within an organisation often produces anxiety about negative repercussions [15]. Despite this discomfort, having ongoing conversations about racism is promoted as an essential part of making progress towards improving the health of affected communities [16].

### Double-loop learning in LEAD

Given that LEAD aimed to implement lasting changes in organisational cultures and processes, the strategies and supporting activities were structured to support ‘double-loop learning,’ that is, an iterative learning process that serves to support changes in organisational norms through questioning assumptions. In double-loop learning, the first loop utilises set goals or rules to make decisions, whereas the second loop is used to question the underlying assumptions of the model [17].

Where ‘single-loop learning’ allows organisations to solve specific and defined problems, Argyris [18] suggests that double-loop learning is necessary for more drastic organisational changes as it allows for deeper reflection and incorporates ongoing feedback to reorient organisational processes towards stated goals and values [18, 19]. Moreover, reflexivity and critical engagement with themes of race and racism are necessary at both individual and organisational levels in order to bring about anti-racism change within organisations [11]. In the case of LEAD, an approach that centred ongoing reflection would therefore be relevant to both elements.

Double-loop learning is particularly relevant in relation to themes that carry some element of perceived risk and where there is a mismatch between an organisation’s stated values and its manifest actions [19], both criteria present in the LEAD programme. This is because, in such cases, the underlying organisational systems that have created the problem need to be addressed through the input of valid information, strengthening trust, and accountability and ongoing monitoring. These requirements aligned with the continued input of evidence provided by the LEAD evaluation taking place concurrently with implementation.

## Methods

One rural and one metropolitan LGA were selected to participate in LEAD based on high levels of racial and ethnic diversity; they were not selected due to particularly high levels of racism in comparison to other Australian communities. The rural LGA has a population of approximately 60,000, 3.4% of which identify as Indigenous and 19.2% of which were born overseas. This LGA is located approximately 200 km from Melbourne. The second LEAD LGA is an outer suburban area of Melbourne. Its population is approximately 155,000 with 0.7% of its population identifying as Indigenous and 38.3% having been born overseas. The Victorian LGA, ranked 1 using the Socio-Economic Indexes for Areas (SEIFA), is the most disadvantaged; an LGA ranked 79 is the most advantaged in the state. The rural LEAD LGA and the metropolitan LGA have a SEIFA ranking of 25 and 42, respectively.

Ethics approval to conduct this study was received from Melbourne University Human Ethics Sub-Committee (HESC) on 27 January 2010.

### Procedure

The LEAD evaluation was structured to maximise the engagement of communities affected by racism and build capacity where possible. The evaluation team included an Aboriginal academic who contributed to evaluation design, development of evaluation tools and resources, and data analysis. Consultation with Aboriginal and CALD communities regarding evaluation tools and survey design was undertaken and community evaluators were trained and employed to administer surveys. Feedback to and engagement with community organisations was also undertaken throughout LEAD as research results became available.

#### Interviews and discussion groups

Key informant interviews with individuals who had participated in LEAD implementation (33 interviews; *n* = 36) sought to examine the perceived relevance of the LEAD programme to organisational priorities, organisational support and leadership with regards to reducing racism, methods of communication, and intermediate outcomes. In addition to these topics, discussion groups and interviews with members of LEAD governance structures (8; *n* = 17) covered the effectiveness and role of governance structures and partnerships within LEAD and mechanisms to continue the implementation of strategies developed from LEAD implementation. Aboriginal and CALD community members or representatives from community organisations were interviewed both as implementing partners and as members of governance structures.

Interviews and discussion groups were recorded and transcribed. Directive content analysis was used, as described in Hsieh and Shannon [20], in order to code the data for content and themes. Coding categories were identified a priori to explore the key issues outlined above regarding LEAD governance structures and partnerships and organisational change. Additional categories were developed to describe issues that emerged from the data. Directed content analysis was selected as the study aims to examine the strengths and weaknesses of the partnership model in facilitating the uptake of evidence in health policy and practice and to explore related themes of organisational change. The use of directed content analysis is in line with Hsieh and Shannon’s indications that this method is appropriate when the goal of the analysis is to build or expand upon existing theory or research.

#### LEAD programme delivery and implementation

Throughout the LEAD programme, data were collected on programme processes, reach and implementation in each of the settings. This information was collected by LEAD council staff and provided to the evaluation team.

Three reflection sessions were held with members of the LEAD Operational Group and an external evaluator to capture key lessons and challenges throughout the programme. Summary reports were collated and distributed to the attendees following each session and highlighted processes around programme development, partnership formation and LEAD governance at each stage of the programme.

**Fig. 2:**
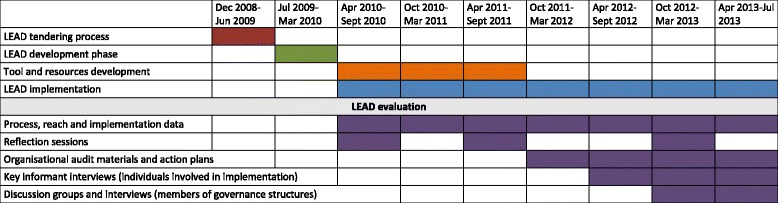
LEAD programme development, implementation and evaluation timeline

Organisational audit materials and action plans were collected by LEAD council staff from LEAD councils, workplaces and schools, and provided to the evaluation team. Analysis of these data entailed assessment of pro-diversity practices and policies evident in the organisations at the time of the audit and subsequent examination of planned and completed actions at the end of LEAD implementation (Fig. 2, Table 1).

**Table 1:** LEAD data collection summary

Key informant interviews (individuals involved in implementation)	33 interviews; *n* = 36
Discussion groups and interviews (members of governance structures)	8 groups/interviews; *n* = 17
Reflection sessions (number of sessions)	3
Organisational audit materials and action plans (number of organisations)	
Councils	2
Workplaces	1
Retail	1
Schools	8

## Results

The structure of LEAD, with its emphasis on collaboration between academics, practitioners and policymakers, had positive implications for the execution of the programme. The implementing councils, funding bodies and other stakeholders were represented at multiple levels through different elements of the governance structure, allowing various pathways for input into the design, implementation and evaluation phases of the programme.

The evaluation team worked closely with the implementing partners throughout the LEAD programme to provide advice and expertise in relation to the available evidence, collect data regarding implementation processes, examine outcomes arising from individual strategies as well as the programme as a whole, and disseminate findings as necessary within the settings as well as externally. The provision of technical expertise at all phases provided an efficient pathway to promote the uptake of available evidence from design through to programme implementation. Discussions between the evaluation team and other partners through the governance model assisted in the development of programme strategies that were evidence-informed and acceptable to all those involved.

“*I think from my… from where I'm sitting, you know, as a manager of LEAD sitting in here, it was that very clear, that very concise, information that we could constantly go back to. That was really helpful. And I think having Melbourne Uni, VicHealth, alongside us to always use a reference point to say, ‘Well, you know, this situation is happening now. What would you recommend or what do you know?’ That was always incredibly helpful.*” (Manager, Metropolitan Council)

Within LEAD settings, the evaluation team and LEAD Project Officers situated within the councils worked in conjunction to provide necessary information to the appropriate internal audiences. The mainstream organisations that were the target of programme intervention varied in terms of their understanding of racism, belief that racism was a problem in their communities, and belief that their organisation could be perpetuating racism or play a role in reducing it. The LEAD aim of improving community health by addressing racism was not one that necessarily made intuitive sense to staff members and decision-makers within the organisations. The link between racism and health was not always apparent, and working with organisations to reduce racism, rather than working with affected communities to cope with racism, was an unfamiliar approach. Consideration, therefore, needed to be given to how to craft these messages for organisational partners. Particularly at the beginning of implementation, a high level of engagement was undertaken by both the evaluation team and the Project Officers. The role of Project Officer as a dedicated, funded position was key to maintaining ties between the evaluation, implementation and managerial/leadership roles in the LEAD programme and facilitating both communication and action.

A series of meetings, discussions and presentations undertaken by the evaluation team and the Project Officer at the beginning of LEAD implementation served to strengthen understanding of the programme itself within implementing organisations. Staff members, including managers, were provided with more information about the rationale behind LEAD, the evidence regarding role of organisations in addressing racism, the link between racism and health, and the possible business and economic benefits of a diverse and harmonious workforce. This involved a substantial time commitment from the Project Officer and evaluation team members. The initial LEAD project launch initiated these discussions, with meetings also being held at the beginning of survey administration, at key points before and during project implementation, and towards the end of the project to disseminate evaluation findings.

“*Because they didn't understand the complexities of it. So they might have seen one aspect of it and just thought, ‘This is nonsense. It's not going to work.’ So that stopped at some point …because, you know, we spent a lot of time explaining the project and explaining the complexities of it…managers would come to me and say those things. So getting managers to understand the complexity of it and the subtleties of it took a little bit of time.*” (Manager, Metropolitan Council)

While the Project Officer was able to report on the day-to-day activities of the implementation process, the evaluation team collated this data to provide an overview of the reach and direction of the strategies undertaken. In addition to supplying ongoing evaluation results, the evaluation team was able to provide evidence from the literature and advise on best practice in response to staff members’ queries about the programme. Feedback from staff was also used to refine tools developed for evaluation that were piloted during implementation, including a pro-diversity organisational audit tool. In this way, circular feedback served to strengthen both implementation and the evaluation process concurrently.

Through fulfilling the need for information across the project timeline, inclusion of evaluation alongside implementation facilitated double-loop learning and shifts in the organisational cultures within specific settings, such as councils and workplaces. These changes were reflected in improved recruitment and employment policies and mechanisms that were put in place to support ongoing implementation of pro-diversity activities after the programme ended. These settings demonstrated increased pro-diversity attitudes as well as reported improvements in employee relations.

“*So really it was a matter of some of the strategies that we found out about in LEAD and what worked for our people as far as communication…We didn’t have any industrial relations issues during that time period when we very well could have so all the new changes bedded down very well and any issues that, issues that were raised by the people they came straight to me and it meant that we were able to resolve them really quickly, which is great.*” (HR Manager, Workplace setting)

The long timeline of LEAD (covering 4 years) facilitated double-loop learning, as it allowed for strategies and approaches to be tested, assessed and amended as needed. Ongoing dialogue between the organisations, Project Officers, the evaluation team and VicHealth during the process of implementing LEAD strategies provided structured opportunities to discuss the organisations’ values regarding diversity, existing policy and practice, and possible discrepancies between the two. Within the LEAD framework, this became part of an iterative process where implementation could be phased in order to build gradually upon successes and adoption of strategies identified as beneficial.

Even with the long timeframe, however, implementation was most successful within organisations that had previously demonstrated some level of engagement with issues around diversity and an understanding of the benefits of an inclusive organisational environment [14]. Additionally, given the intensive time investment necessary for the ongoing processes of dialogue and feedback between the organisations, the Project Officer and the evaluation team, changes were most apparent in organisations that had pro-active individuals who were highly involved in the programme and were able to facilitate and drive change.

The intensive and simultaneous input of effort from multiple organisations also represented a large drain on resources when elements of the programme were unsuccessful. Given that LEAD was a pilot programme, there were a number of approaches that were trialled and found to be ineffective. One such example was the strategy of working within retail organisations to reduce exposure to racism for both employees and customers. While this approach was supported by the theory underpinning the LEAD programme, repeated attempts to invite participation by retailers over a series of months were unfruitful. Retail managers expressed reluctance to participate in LEAD as they did not believe that their organisations fostered racism and because they feared that being involved in an anti-racism programme would be harmful to their reputation.

“*Part of our business – I don’t know if you noticed when you walked through – but there would have been a lot of people in red jumpers that said ‘hello’ to you and that’s just based on ‘You’re a customer; we speak to all our customers equally’ – it’s just part of our culture here. We just didn’t feel that it had any sort of benefit to the team at all.*” (Manager, Workplace setting)

The time and resources invested in this strategy over a few months were considerable. The primary actors affected were the Project Officers in each of the LEAD sites, who engaged directly with the retail organisations to little avail. As part of the initial steps, the evaluation team conducted surveys with the tentative retail partners, as well as subsequent data analysis and producing preliminary reports. The surveys were undertaken in all LEAD settings and served to establish baseline staff attitudes and experiences as well as forming the basis for discussion and development of LEAD strategies in the setting. The funding body, which played a key role in programme development and management, also expended significant time in supporting and encouraging the Project Officers in their attempts to work with the retail setting, which was ultimately futile.

Given the complexity of the LEAD model and the number of actors operating simultaneously, maintaining efficient communication and clarity of roles was a challenge. Some degree of diffusion around the roles of the funding bodies, implementing partners and evaluation team was built into the model itself. The funding bodies provided advice and guidance on programme development and implementation, as did the evaluation team, while the implementing partners were also key in evaluation data collection and dissemination. With these boundaries being somewhat unclear, being able to determine which individuals and organisations needed to be aware of a particular piece of information without overwhelming all parties with irrelevant communication was sometimes difficult. The process for resolving conflict between implementing partners, the evaluation team and the funding partners, including who had the final say in making which decisions, was also not always transparent. In these instances, the multiple avenues for discussion between partners represented by the governance structure facilitated dialogue, which normally led to an agreed-upon solution. In order to clarify expectations, within the first few months of LEAD, all partners participated in constructing guidelines regarding communication, roles, dissemination protocols and intellectual property. The development of these guidelines supported a thorough examination of needs and expectations across the wide range of stakeholders represented by the LEAD partners and ultimately led to more efficient collaboration.

## Discussion

There is an outstanding need for rigorous research examining which strategies to reduce racism are likely to have the strongest impact on the health outcomes of minority communities in order to underpin effective anti-racism policy [9]. It is also not just policy but implementation that needs to be informed by appropriate research and process evaluation and feedback to ensure that anti-racism programmes are being conducted appropriately [21]. In the case of anti-racism interventions, monitoring and evaluation should be conducted alongside implementation, due to evidence that anti-racism interventions can cause more harm than good if poorly designed. For example, target audience members may become more entrenched in racist views due to feeling challenged, defensive or resentful if the intervention is not appropriately managed [22]. Poorly conducted cultural training may reinforce a superficial, stereotypical or ‘essentialised’ view of ‘Indigenous culture’ without incorporating reflection on audience members’ own identity and cultural beliefs or inequitable power structures, thus further entrenching oppressive dynamics [23].

The LEAD governance model was underpinned by a strong emphasis on partnership-building and collaborative priority setting. The structure of LEAD, with its collective efforts towards developing effective anti-racist practice, ongoing engagement, and shared knowledge and resources can be understood as a Community of Practice spanning sectors and organisations [5]. The Community of Practice framework lends itself to visualising how partnerships between researchers, policymakers and practitioners can facilitate the productive interconnection between evidence generation and the uptake of evidence into policy and practice [6]. In this framework, the participation of community members is geared towards engagement, learning and the creation of knowledge that will serve to improve the practice around which the group has formed [5, 6]. In the case of Canadian tobacco control, the use of Communities of Practice was shown to be effective in bringing together health promotion research, practice and policymaking [6]. In considering the structural dimensions described by Mitchell et al. [2], the LEAD partnership exhibited an emphasis on the needs established by decision-makers, with research playing a supporting role to identified priorities at each stage in the process. In this way, stakeholders played an active role in the development and design of the evaluation and had significant investment in the research, as it directly impacted on the implementation and ultimate success of the programme itself. The inclusion of a number of partner organisations from different sectors in the development and design of LEAD also ensured that racism would be considered and addressed from a variety of perspectives. This ongoing exchange between researchers, implementers and other stakeholders formed the basis of translating existing and emerging evidence into effective practice within the LEAD programme.

Griffith et al. [24] characterise institutional racism as an embedded and complex problem, necessitating a focus on system, multi-level change. Moreover, given that institutional racism is born out of power dynamics that are not always evident, Griffith et al. [24] posit that an effective intervention must incorporate the time and space needed for reflection and examination. These themes were strongly present in the LEAD experience. Echoing Griffith et al.’s ‘dismantling racism’ approach [24], LEAD implementation focused on the reorganisation of policies and processes and provided opportunities for individual-level critical engagement and reflection through training workshops. The ‘dismantling racism’ approach highlights that monitoring and evaluation is necessary in order to keep track of progress and barriers; however, little detail is given regarding how this evaluation is to take place, or how such data should be used to improve anti-racist practice.

Within LEAD, the iterative processes of monitoring, evaluation and adaptation, drawing heavily from an action research approach, strengthened this aspect of ‘dismantling racism’. In order to be effective, Trip [12] indicates the desirability of having action research supported by organisational networks that facilitate knowledge management. Within implementing organisations, the Project Officer served as a facilitator between the organisations’ management teams and the rest of the LEAD partners. This allowed organisations to have access to expertise from a range of different sectors and also enabled stakeholders to be kept abreast of knowledge generated during implementation and evaluation. Changes to policy and practice at the organisational level were able to be strengthened as findings from the concurrent evaluation and existing evidence were used to guide intervention throughout the programme. The experience of LEAD highlights the point that action research depends on participation, collaboration and co-operation, as the changes undertaken as a result of the research will necessarily involve a range of actors [12].

Through fulfilling the need for information across the project timeline, inclusion of evaluation alongside implementation facilitated double-loop learning, as information was provided in a useable and timely way and allowed for implementation to be continuously corrected or adjusted. Supporting the process of double-loop learning was particularly necessary for participating organisations in the LEAD programme, as engagement with the theme of racism was quite challenging and the approach necessitated a shift in the way people thought about racism in general and within their organisation. The practices of reflection and dialogue have been identified as necessary for the deep questioning of held assumptions, the exchange of ideas and development of shared understanding [25, 26]. The structure of LEAD provided external facilitators, such as the Project Officer and evaluation team members, as well as dedicated spaces and mechanisms to allow this dialogue and reflection to occur. As outlined by Rist and Joyce [21], implementation evaluation in LEAD was dependent on close relationships between the evaluators and programme staff, which enabled improved and appropriate adoption of research findings to underpin organisational cultural changes while simultaneously enhancing the development and implementation of organisational anti-racist tools such as auditing and training programmes [21]. However, even with the level of support provided throughout LEAD, it was not sufficient to effectively engage with organisations that had not previously been primed to undertake pro-diversity action.

The time, effort and resources necessary to produce organisational changes were substantial and demanded input from various organisations, including the funding and implementing partners, the evaluation team and the target organisation itself. The time necessary to refine evaluation, feedback and implementation processes indicates the usefulness of long-term collaboration models between researchers, implementing bodies, funding bodies and other stakeholders, as well as more explicit strategies on how to manage complications when they arise. Baker et al. [27] describe the ‘maturation time’ and ‘transition periods’ for academic/practice/community research partnerships, each of which may be a time of negotiation, adjustment and recalibration within the partnership. Similarly, Butterfoss et al. [28] outline the ‘stages of development’ for cross-organisational coalitions for prevention and health promotion as formation, implementation, maintenance and the development of goals or outcomes, and suggests that each stage requires a different set of strategies for efficient progression. In the case of LEAD, the needs of the partnership in the initial formation and planning stages [27, 28] were considered at length. As part of the purpose of LEAD was to trial this new partnership model, care was taken to clearly lay out the purpose of the partnership, how the different elements of the governance structure would operate and who would be represented in each during the formation phase. During the implementation phase, as referenced above, agreements were developed regarding communication, intellectual property and other points. As the partnership progressed, strategies such as reflective group sessions, ongoing meetings and flexibility in partner responsibilities supported the collaboration through the maintenance phase, particularly through challenges such as staff turnover and shifts in organisational leadership. Nevertheless, simultaneous involvement of multiple stakeholders and organisations proved to be difficult to manage at points. Partner organisations working in conjunction expended significant amounts of time when elements of the programme were difficult or progressing poorly. In particular, it was a delicate balance to organise the time of partner organisations to provide support to implementers in complex situations while also minimising duplication of effort and ultimately making the decision to change tactics. Developing successful and efficient ways of working together – clarifying communication protocols and partner roles – took time and investment of behalf of each of the partners. Awareness of these stages and understanding of the associated tasks and processes may support smoother shifts through the life of such collaborations.

### Implications

The LEAD experience provides a number of key lessons for practitioners, policymakers and programme implementers in health promotion. The model brought together concepts from the Communities of Practice framework and action research practice to enable the simultaneous engagement of a range of stakeholders centred on improving practice. The partnership model supported both the generation of evidence guided by the needs of practitioners and implementers and the uptake of new evidence into programme implementation and policy. The central facets of the model – cross-sectoral involvement, governance structures representing organisations and communities at various levels and multiple avenues for input and shared knowledge exchange – should be considered in future approaches to build stronger connections between the generation and utilisation of evidence in policy and practice.

Within LEAD, double-loop learning as a way of promoting organisational change was supported by the use of evaluation undertaken alongside programme implementation, guided by action research principles. These approaches reinforced each other as the cycle of planning, implementing, evaluating and feedback allowed for the ongoing reflection necessary to question and change underlying organisational assumptions. Moreover, the support lent to organisations by way of the LEAD governance structures and Project Officer provided increased avenues for information sharing and mechanisms for reflection at the individual and organisational level. LEAD therefore provides a concrete example of how these approaches can be used to address complex problems such as racism and discrimination in a way that maximises the use of evidence. Trip [12] notes that very little of the knowledge produced by action research is published, highlighting the need to make this information publicly available.

Given the time necessary for the development of the LEAD partnership model, working towards organisational change and individual and organisational-level reflection around race and racism, a long timeline was necessary for the programme. Close consideration of the needs and purpose of the partnership and the governance structure in the initial stages and subsequent agreements around processes were supportive to functional operation. However, with the complexity of the model and experimental nature of the programme, difficulties in managing relationships, decision-making and conflict arose with little clarity around how to resolve them. Bringing an understanding of the stages of collaborative partnerships and the differential needs in each of the stages to bear on the maintenance phase of the relationship may help to plan effective strategies to resolve these tensions.

## Conclusions

The LEAD model provides a concrete example of collaboration between implementers, academics and others that successfully bridged the ‘knowledge gap’. The LEAD programme provides an examination of concrete mechanisms for structuring health programmes to enhance ongoing interactions between practice, research and policy. The programme aimed to improve the health of Aboriginal and CALD communities through reduction in exposure to racism and demonstrated success in enabling a high level of uptake of anti-racism strategies within employment and council settings [13, 29].

LEAD highlights the need for evaluation to be considered and embedded from the beginning of complex health interventions. This allows the evaluation to inform and be informed by implementation while it occurs and supports the process of double-loop learning, enabling deeper and more strongly embedded organisational change. At the same time, this integrated and collaborative way of working across sectors raised its own set of challenges around clarity of partner roles, effective use of time and resources, and effective communication. While these difficulties were resolved with the active involvement of all parties, the time necessary to do so lends support to the need for long-term collaborative partnerships and explicit strategies to manage the different stages of such partnerships in order to successfully integrate evidence into community-based health interventions.
